# A Machine Learning Approach to the Detection of Pilot's Reaction to Unexpected Events Based on EEG Signals

**DOI:** 10.1155/2018/2703513

**Published:** 2018-04-10

**Authors:** Bartosz Binias, Dariusz Myszor, Krzysztof A. Cyran

**Affiliations:** ^1^Institute of Automatic Control, Silesian University of Technology, ul. Akademicka 16, 44-100 Gliwice, Poland; ^2^Institute of Computer Sciences, Silesian University of Technology, ul. Akademicka 16, 44-100 Gliwice, Poland

## Abstract

This work considers the problem of utilizing electroencephalographic signals for use in systems designed for monitoring and enhancing the performance of aircraft pilots. Systems with such capabilities are generally referred to as cognitive cockpits. This article provides a description of the potential that is carried by such systems, especially in terms of increasing flight safety. Additionally, a neuropsychological background of the problem is presented. Conducted research was focused mainly on the problem of discrimination between states of brain activity related to idle but focused anticipation of visual cue and reaction to it. Especially, a problem of selecting a proper classification algorithm for such problems is being examined. For that purpose an experiment involving 10 subjects was planned and conducted. Experimental electroencephalographic data was acquired using an Emotiv EPOC+ headset. Proposed methodology involved use of a popular method in biomedical signal processing, the Common Spatial Pattern, extraction of bandpower features, and an extensive test of different classification algorithms, such as Linear Discriminant Analysis, *k*-nearest neighbors, and Support Vector Machines with linear and radial basis function kernels, Random Forests, and Artificial Neural Networks.

## 1. Introduction

Introduction of automated systems in plane cockpits significantly increased flight safety. However, in case of a failure of such systems or occurrence of the situation in which these systems are not able to behave correctly, pilots must instantly and unexpectedly make complex decision [1, 2]. Usually utilization of such supporting systems puts the pilot in a passive role; this introduces an additional challenge in case of issue occurrence that might take place after long period of autonomous flight, because pilot must switch immediately to the active role and cope with complex problems that require quick judgment [3, 4]. In addition, high reliability of autonomy might reduce focus of the pilots on monitoring tasks, thus prolonging the time of context switching [5]. Moreover, introduction of automated processes that controls the plane might reduce orientation in the current state of the flying process resulting in automation surprises [1, 6] and some researchers point out that extensive use of autonomy systems might even decrease flying skills of the pilots [7].

On the other hand, performance of pilots and thereby safety of flights can be greatly improved and increased thanks to cognitive cockpit solutions [8, 9]. These systems provide an adaptive support for decision processes and control tasks involved in aircraft operations. Such solutions can be highly profitable both for military and passenger flights. One very critical feature of such systems applies to the elimination of human related errors and prevention of disasters that may result from them. A prominent solution to that can be found with Man Machine Interaction systems such as Brain Computer Interfaces (BCI) [10]. These systems are capable of monitoring and interpreting of brain activity for computer or prosthesis control, rehabilitation, and other purposes. Such approach comply with the Human-Centred-Automation concept [11] in which human interacts with the controlled system in an efficient way that can be further improved through supporting of the cockpit logic with information about brain activities. Another interesting application of BCI based systems might involve an assessment of pilots' mental state and capabilities executed in before-flight-phase as well as during pilots' training process, for example, in order to train pilots that have tendencies to be less alert. Such systems can be used, for example, by recruitment agencies to evaluate the natural predispositions of pilots.

BCI systems are often based on electroencephalographic (EEG) signals [12]. EEG signals are recorded by measurement sensors that are placed in specific locations over the scalp. These sensors are referred to as electrodes. Due to characteristics of EEG signals that make them highly susceptible to noise and artifact disturbances, differential measurement configurations (uni- or bipolar) are commonly used. As a result of EEG measurement the* electroencephalogram* (EEG) is obtained. A few characteristic frequency bands are often mentioned in the context of EEG: delta (below 4 Hz), theta (4–8 Hz), alpha (8–12 Hz), beta (12–28 Hz), and gamma (over 30 Hz) [13–15]. It is worth mentioning that the frequency limits of specific waves are conventional as there is no proper way of determining their exact values. Delta brainwaves are commonly associated with deep sleep [13]. Theta activity is present during states of drowsiness. Interestingly, theta activity has been also observed during cognitive visual processing [16]. The alpha activity occurs during states of wakeful relaxation or tiredness and can be induced by closing eyes [13, 15]. Although being commonly attributed to states of relaxation, these rhythms may increase during some attention tasks [15]. Beta waves are associated with normal waking consciousness, alertness, and an active concentration [13, 17]. The role of gamma waves remains an active topic of a research. The reproducibility of the conducted EEG research is ensured by utilization of some universally accepted standards of electrode placement and annotation [13]. Among most popular systems mentioned can be standard* 10-20* as well as its extensions such as* 10-10* and* 10-5* [18, 19].

In this research use of EEG signals recorded with inexpensive device (Emotiv EPOC+ headset) is evaluated for the purposes of cognitive cockpit applications. Precisely, the possibility of discrimination between two states of event-related activity is tested: (i) brain activity related to idle but focused anticipation of visual cue (pre-event) and (ii) reaction to that cue (event-related).

## 2. Materials and Methods

### 2.1. Emotiv EPOC+ Headset

Emotiv EPOC+ Headset device was used for the purpose of recording EEG data during the experiment. In a study that examined the sensitivity of few inexpensive, wireless, and/or dry (no gel) electrode EEG systems, Emotiv has proven to perform well (compared to a traditional, research-grade EEG system) in tasks concerning measurement of alpha brain activity and Visual Steady-State Response (VSSR) [20]. Due to setup problems authors of that work were not able to provide evidence to support the use of Emotiv in paradigms that rely on time-locked events. However, some reports of use of Emotiv EEG systems in such tasks are available [21].

The recorded signals useful bandwidth is in 0.16–43 Hz range and is sequentially sampled with frequency 128 Hz and 14-bit (1 LSB = 0.51 *μ*V) resolution. EPOC+ has built in digital 5th-order Sinc filter and notch filters at 50 Hz and 60 Hz [22]. 14 EEG channels available in Emotiv EPOC+ Headset are compatible with the following electrodes of the international 10-10 montage system: AF3, F7, F3, FC5, T7, P7, O1, O2, P8, T8, FC6, F4, F8, and AF4, with references in the P3/P4 locations.

**Figure 1:**
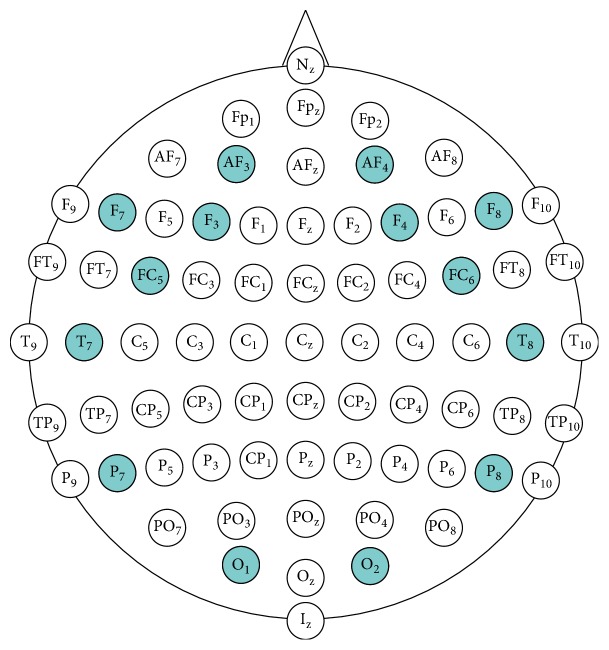
Positions of electrodes in the standard* 10-10* electrode montage system (own source based on [19]).

The placement of EPOC+ electrodes in the 10-10 configuration was marked in Figure 1 [19].

Some special precautions were undertaken to reduce the contamination of the data by artifacts related to muscle movements that occur, for example, during motor actions of limbs, head repositioning, or blinking. All subjects were seated in a comfortable position and instructed to limit their movements as much as possible. Additionally, time segments which were used in this research were visually inspected for the presence of artifacts. Trials that were assessed to be too contaminated were removed from the analysis.

### 2.2. Flight Simulators

**Figure 2:**
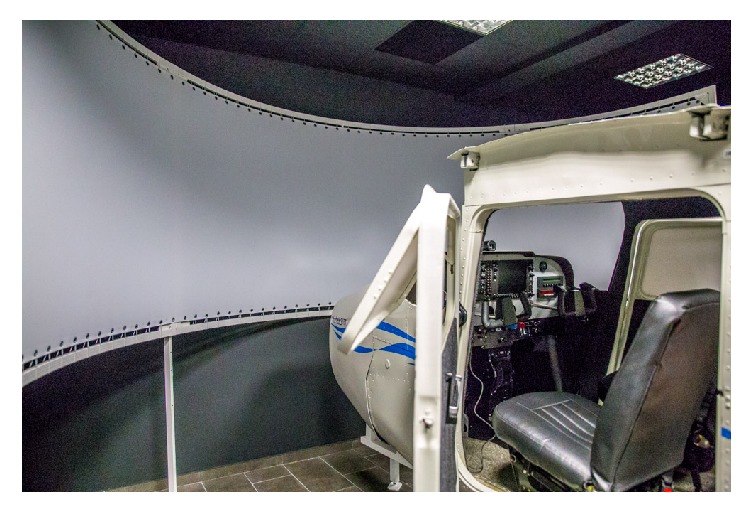
Interior of used flight simulator (cockpit) and a simulation screen.

Flight Navigational Procedure Training II (FNPT II) class simulator that passed QTG tests was utilized during data acquisition phase. Simulator represents Cessna 172RG plane model. It consists of fully enclosed full size cockpit that faithfully reproduces internals of Cessna 172RG equipped with glass cockpit. It is characterized by 180 degree panoramic view of the environment that is generated by three projectors. Simulator is located in an especially designated room (Virtual Flight Laboratory located at Silesian University of Technology), without any windows and with black walls thus no external stimulus can reach the pilot. In addition air temperature is controlled so every experiment can be conducted in the same conditions. Presented in Figure 2 is an interior of the cockpit of used simulator.

### 2.3. Experiment Description

Through the experimentation phase, measurements of a human brain activity during simulated session of short haul flights with activated auto pilot were acquired. The purpose was to obtain brain response to randomly displayed visual cues that were presented on the main screen of the simulator.

Participants were selected from the group of people aged between 20 and 35. All participants claimed that they were well rested before the session, and all of them gave consent to utilization of outcomes obtained during the experiment for the purpose of scientific researches. During experimentation phase 10 people (all males) were examined. Every experimental session started at the same time of the day around 12:00 (noon). It was ensured that through the experiment no external factors had influenced its participants. Each session took around 1 hour. Experiments took place in FNPT II class simulator. Participants had to observe cockpit instruments as well as scan the surrounding of the plane so to behave as pilots during regular flight. They were instructed to stay focused and maintain awareness in order to be able to instantly react to the appearance of visual cue by pressing of a specific button. The placement of button was chosen to minimize the time required for reaction to visual cue by restraining any additional movements of pilots body besides their fingers.

In order to maintain consistency between successive experimental sessions simulated flight on the route between Frankfurt and London was registered. The same section of the flight was presented to each participant of the experiment. The terrain over which flight took place and cockpit instruments were recorded. During this flight auto pilot was activated. Flight took place at the average altitude of 6,000 feet. In order to simulate flight with auto pilot activated, take off and landing were removed from registered material. Moreover, whole flight that was presented to the participants took place over land. Importantly sound of engines was also generated in the cockpit.

Visual cues were displayed randomly with normal distribution characterized by *μ* = 2.5 minutes and *σ* = 1 minute. Variance was introduced in order to prevent habituation of human brain to regular patterns. In addition, for each pilot distribution of visual cues in time was the same. Visual cue was represented by solid grey-colored box that overlap 75% of the main simulator screen that is responsible for displaying of the terrain.

Bioethical committee of The Jerzy Kukuczka Academy of Physical Education in Katowice consent was obtained for conduction of this type of experiment.

### 2.4. Class Definition

For the purpose of conducted experiment two classes of mental activity were defined. Since the phenomenon analyzed in this research is related to an appearance of some visual, the problem is in fact a problem of event-related activity analysis. Therefore, the following class definitions were adopted:

- *Pre-event*: a focused anticipation of visual cue
- *Event-related*: activity related to reaction to the visual cue

**Figure 3:**
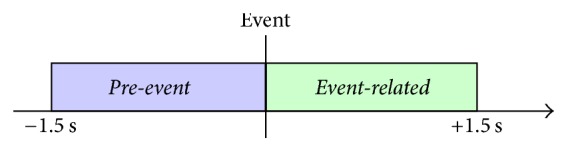
Concept of* pre-event* and* event-related* class trials extraction (own source).

The* pre-event* trials were calculated from time window of 1.5 s length containing samples directly preceding the appearance of visual cue. Trials of* event-related* class were determined analogously, from all trials that followed the presentation of cue and that belonged to 1.5 s long time window. As a result one trial of each class was obtained for each event. A concept of* pre-event* and* event-related* class trials extraction is presented in Figure 3.

### 2.5. Spatial Filtering

To improve and enhance discriminative characteristics of signals that could have been degraded by volume conduction related effect, the* Common Spatial Pattern* (CSP) has been used in this research [10]. CSP is a technique used for analysis, decomposition, and transformation of multichannel EEG recordings containing two classes of different mental activity. It is a popular method of spatial filtering, commonly used in Brain-Computer Interface applications. It has proven to be especially effective with logarithmic bandpower used as a feature describing the brain activity. Although it is most commonly associated with motor imagery, it might prove to be valuable approach to implement it in our research in a task related to visual processing. Many works show the superiority of CSP over classical spatial filtering methods such as Surface Laplacian, Common Average Reference, ICA, and others, thus justifying the choice of CSP in this research [12, 23]. Variance of transformed EEG signals is maximized for trials from one class and simultaneously minimized for examples from another class. For that purpose transformation matrix *W* ∈ *ℝ*^*N*×*N*^ is provided (*N* denotes the number of measurement channels). Matrix *W* consists of column-wise of optimized spatial filters that correspond to its eigenvalues. More detailed description of this problem can be found in [10]. In general, to avoid overfitting only few pairs of filters from both ends of eigenvalue spectrum carrying a discriminant information are used. In this work, 3 best CSP filter pairs from each frequency subband were taken into consideration for each subject.

Let us assume that *M* correspond to length of single trial *X* ∈ *ℝ*^*M*×*N*^ of EEG phenomena. Then, spatially filtered signal *X*^CSP^ ∈ *ℝ*^*M*×*N*^ of a single trial *X* can be calculated with the use of projection matrix *W* as presented in the following:

(1) $$\begin{array}{c} X^{CSP}=W^{T}X. \end{array}$$

### 2.6. Bandpass Filtering

It is a well known fact that performance of the CSP method depends highly on the frequency bandwidth in which signals are analyzed. Therefore, signals must be properly band-pass filtered before applying CSP to them. Selection of appropriate frequency range is therefore a critical and difficult task [10]. Many solutions to that problem have been proposed; however one of the most prominent approaches up to date remains to be the Filter Bank Common Spatial Patterns (FBCSP) [23]. In this approach signals are first filtered into *F* multiple frequency subbands. Then, CSP is applied to each of the filtered signals independently. A fixed number of *P* filter pairs is taken from each band to form a general set of features. To avoid overfitting a feature extraction procedure must be then applied. For the purpose of this article signals will be bandpass filtered into the following ranges corresponding to specific brainwaves: delta (1–4 Hz), theta (4–8 Hz), alpha (8–12 Hz), low beta (12–16 Hz), middle beta (16–20 Hz), middle-high beta (20–24 Hz), high beta (24–28 Hz), two frequency ranges related to lower gamma frequencies, respectively, gamma 1 (32–36 Hz) and gamma 2 (36–40 Hz), and 8–30 Hz range that is commonly related to planning of motor movement that will be referred to as motor.

For the purpose of bandpass filtering of EEG data a Kaiser Window Finite Impulse Response (FIR) band-pass filter constructed of 466 coefficients was used. Since the analysis was to be performed offline (no requirement of causality of used algorithms) a zero-phase (nondelaying) filter could be applied. This operation was implemented by applying a recursive filter to the original signal both forward and backward in time [24]. Let *x* ∈ *ℝ*^*M*^ be a recorded, discrete signal consisting of length *M* and *h* be the impulse response of the recursive filter. The output *v* ∈ *ℝ*^*M*^ of filtering operation performed on *x* is calculated as in

(2) $$\begin{array}{c} v=h*x. \end{array}$$

If *x*(*i*) (*i* = 1,…, *M*) denotes a discrete sample o *x*, then the operation of flipping the signal can be defined as in the following [24].

(3) $$\begin{array}{c} \text{flip}\left(\;x\left(\;i\;\right)\;\right)=x\left(\;M-i\;\right),\;\forall i\in Z,\;\;i<M. \end{array}$$

The flip operator reverses the order of samples of a discrete signal *x* [24]. Considering the above definitions the output of forward-backward filter *y* ∈ *ℝ*^*M*^ can be calculated as presented in the following[24].

(4) $$\begin{array}{c} y=flip\left(\;h*flip\left(\;h*x\;\right)\;\right). \end{array}$$

### 2.7. Feature Extraction

A logarithm of the variance of signal's amplitude is a very common feature used for the description of EEG signal's power [10, 25]. As mean value of bandpass filtered EEG signal is close to 0, its power is in fact equivalent to its variance. The normalization of the feature distribution is obtained by an application of logarithm operation [25].

The band power features were used for the analysis of brain activity during the experiment. They were calculated from a spectrally and spatially filtered signals, individually for each measurement channel from all samples that belonged to class-specific time window (either* pre-event* or* event-related*).

### 2.8. Feature Selection

After creating a bank of filters by bandpass filtering of EEG signals into *F* = 10 subbands and applying a CSP transformation to each subsignal a set of *K* = *F* × *N*_ch_ = 140 features was obtained (*N*_ch_ = 14 denotes the number of measurement channels of EPOC+). The most discriminative subset of features was selected by ranking all features based on the mutual information (MI) criteria. MI of features describing two categorical classes (*pre-event* and* event-related* in this work) represents the dependency between these features. If samples of a given feature are independent for defined classes their MI will be equal to zero. The higher the calculated MI values, the less discriminative the features. Mutual information for a discrete variables was obtained with nonparametric methods based on entropy estimation from *k*-nearest neighbors distances [26–28]. In this work *N*_sel_ of best features from ranking (with biggest difference in MI) were selected. In implemented feature selection approach, feature ranking was created only utilizing a features from a training data independently from classifier. However, a number *N*_sel_ was tuned individually for each validation session on the basis of classifier performance on the cross-validation data. Therefore, an implemented method cannot be unambiguously described as a filter approach. A detailed description of the whole feature selection and machine learning pipeline implemented in this research can be found in Section 2.9. Use of MI-based feature selection methods has been proven to yield highly satisfactory results in filter bank approaches to EEG signal processing [23].

### 2.9. Data Classification

To properly evaluate an accuracy of proposed model a stratified modification of leave-one-out procedure was implemented. In this approach one sample from each class is being used as the testing set. Precisely, one trial from* pre-event* and one trial from* event-related* class related to the same event are selected to form a two-element test set. Remaining samples are used to create a training set. Described validation procedure allows taking into consideration the chronological order of the trials. Proposed approach resembles a real life case where training trials used for the calibration of pilot aiding system are recorded consequently during specified time frame. Such examples will share some common characteristics that might differ for trials recorded in later stages (i.e., during the operation of the system). The resemblance of the proposed procedure of data partitioning to the real applications is a significant advantage over random choice of trials. This training set is used not only to train given classifier but also to determine the CSP transformation matrix *W* and for the purposes of feature selection. This is dictated by the fact that use of test data for that purpose would lead to overfitting of the model and result in biased estimation of model accuracy. Described steps are repeated for every event that is available for each subject. Final accuracy of proposed model is obtained from the mean of all accuracies achieved in particular cross-validation stages.

In this research an extensive test of different classification algorithms, such as Linear Discriminant Analysis (LDA), *k*-nearest neighbors (kNN), Support Vector Machines with linear (SVM_LIN_) and radial basis function (SVM_RBF_) kernels, Random Forest (RF), and Artificial Neural Networks (NN) was performed. A standard pipeline of machine learning processing implemented for each classifier begins with extraction of bandpower features, normalizing their distribution by application of logarithm transformation, removal of their mean, and scaling the variance to unitary. Such standardization of features is often required for many machine learning estimators to perform in a satisfactory way. The next step involves ranking the features by their MI and preliminarily selecting 9 of them for the stage of classifier tuning. The final number of features *N*_sel_ is selected during the process of machine learning estimator fine tuning. For that purpose a cross-validated grid search strategy was utilized. In this approach, all possible combinations of hyperparameters that were specified by the user are tested and the combination that allowed achieving the best accuracy is selected. For that purpose the training data is furtherly divided into two subsets: one used for training and the other for cross-validating tested parameters. That was achieved with the 3-fold cross-validation. After the best combination of hyperparameters is selected, the estimator is refitted with them on the whole training dataset.

Presented below are brief summaries of each tested classification algorithm together with descriptions of sets of hyperparameters used during the tuning process. For each subject and each validation session classification model was created using full training dataset with selected best hyperparameters and used to obtain a classification accuracy on the test data. Achieved results and comparison of classifiers performances are presented in Section 3.

#### 2.9.1. Linear Discriminant Analysis

LDA is a simple classifier with a linear decision boundary, obtained by fitting class conditional densities to the data and using Bayes' rule. It is a parameterless estimator that did not require any fine tuning. Creating a model with LDA requires the estimation of class covariance matrices. However, in situations where the number of training examples is small compared to the number of features the empirical sample covariance is a poor estimator. In such scenarios use of shrinkage can improve estimation of covariance matrices. The level of shrinkage can be controlled by specifying the shrinkage parameter. For a 0 value of no shrinkage, the empirical covariance matrix is used. For a value of 1 the diagonal matrix of variances is used as an estimate for the covariance matrix. The optimal shrinkage parameter was obtained following lemma introduced by Ledoit and Wolf [29].

#### 2.9.2. *k*-Nearest Neighbors

kNN is a distance based classifier capable of solving nonlinear machine learning problems. In this work the number of neighbors was selected from the range 1 to rounded value of (4(*N*_*e*_ − 1)/3) − 1, where *N*_*e*_ is a number of events that occurred during the experiment. For the distance calculation the Minkowski metric was used. The power parameter of this metric was selected from the range 1–5. The points in each neighborhood either were considered with uniform weights or have been assigned weights proportional to the inverse of their distance from the analyzed point.

#### 2.9.3. Support Vector Machines with Linear Kernel

SVM_LIN_ belongs to a group of supervised learning methods used for classification (or regression). These methods are quite effective in cases, such as the one presented in this article, where dimensionality of feature space is greater than the number of examples. However, if the number of features is much greater than the number of samples they are prone to overfitting.

The best value of penalty parameter *C* of the error term was selected from the set of values evenly spaced on the logarithmic space from −4 to 50 with step 5. During the grid search parameter optimization it was determined for each session whether to use the shrinking heuristic or not. Tolerance for stopping criterion was selected from the values 1*e* − 1, 1*e* − 3, 1*e* − 5. The calculations could be also terminated if the upper limit of iterations 1*e* + 5 was reached.

#### 2.9.4. Support Vector Machines with Radial Basis Function Kernel

SVM_RBF_ is a SVM algorithm that thanks to the use of nonlinear kernel is capable of solving more complex problems. Additionally, utilization of RBF kernel can help avoiding overfitting in situations where dimensionality of feature space is greater than the number of examples.

The RBF kernel coefficient's value, as well as the best value of penalty parameter *C* of the error term, was chosen during the fine tuning stage from the set of values evenly spaced on the logarithmic space from −3 to 20 with step 2. During the grid search parameter optimization it was determined for each session whether to use the shrinking heuristic or not. Tolerance for stopping criterion was selected from the values 1*e* − 1, 1*e* − 3, 1*e* − 5. The calculations could be also terminated if the upper limit of iterations 1*e* + 5 was reached.

#### 2.9.5. Random Forest

RF is an ensemble estimator that fits a number of decision tree classifiers utilizing variously subsampled examples from the training dataset in order to improve the accuracy and avoid overfitting. The final classification is obtained by taking the majority vote of all decision trees. In this work, the size of subsampled training data is always the same as the original input sample size. This was maintained by the utilization of sample bootstrapping (sampling with replacement). The nodes of each decision tree were expanded until all leaves were pure or until all leaves contain less than some individually tuned minimal number of samples per each split. This number was selected from the set of evenly distributed number (with step 3) from range 1 to 15. The quality of splits could be evaluated with either using the Gini impurity or entropy criteria. The number of trees in the forest was chosen from the set of evenly distributed number from range 1 to 100 with step 5 during the grid search hyperparameter tuning. The RF classifier creates new training subsets with bootstrapping. This approach is often referred to as bagging. As a result, a part of the training set remains unused and can be utilized for the task of the generalization error estimation. During that hyperparameter tuning it was also determined whether or not to use out-of-bag samples to estimate the generalization accuracy. It must be noted that due to the fact that RF is a tree-based classifier it is capable of ranking the features itself. Each feature can evaluate how it improves the chosen quality of split. Nodes with the greatest decrease of said measure are most discriminative. Therefore, by restraining (pruning) trees below a particular node, a subset of the most important features can be created. The number of features to consider was fine tuned from range 1 to 140 with step 10 during the grid search hyperparameter tuning.

#### 2.9.6. Artificial Neural Networks

Feed Forward Artificial Neural Networks with one hidden layer were evaluated. During initial phase of tuning process NN with various numbers of neurons in hidden layer (in the range 1 to 100) and ReLU activation function were tested. LBFGS solver was exploited for the training process. The purpose was to determine the smallest NN structure that is characterized by the best recognition properties. Results pointed out that the best accuracy was delivered by NN with 4 neurons in hidden layer. Therefore, this structure was selected for the second phase of NN tuning. Due to the fact that the results of NN training process are highly dependent on initial weights between neurons within NN structure, process of NN training was repeated independently 100 times. At the beginning of each training scenario NN weights were initialized with random values. After execution of the second phase of the tuning the best NN were selected.

## 3. Results and Discussion

**Figure 4:**
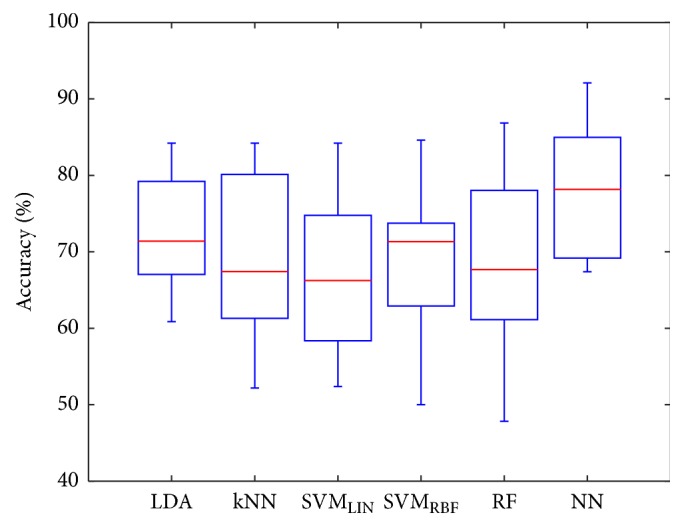
Comparison of classifier performance obtained for all subjects.

In Figure 4, performances of classifiers have been compared and visualized with the help of box plots. Additionally, accuracies achieved by each of the evaluated classifiers for each subject obtained from the validation procedure described in Section 2.9 are presented in Tables 1–6. In Table 7 the distributions of results across all experimental sessions for each classifier are summarized. For that purpose mean accuracy *μ*, standard deviation *σ*, first quartile *Q*_1_, and third quartiles *Q*_3_ were calculated.

The visual inspection of box plots presented in Figure 4, as well as the analysis of distributions presented in Table 7, suggests that the performance of a Neural Networks might be significantly better than that of other algorithms. In order to evaluate that hypothesis a one-way analysis of variance (ANOVA) has been performed. The tested hypothesis was that the means of all accuracies obtained for each subject by different classifiers are the same against the alternative hypothesis that the populations means are not all the same. High *p* value obtained from said ANOVA test (*p* = 0.2708) might suggest that differences in mean accuracies of all classifiers are not statistically significant. This however might be attributed to the small size of the populations. One versus one comparison of Neural Networks against LDA, kNN, SVM_LIN_, SVM_RBF_, and RF returned, respectively, following *p* values: 0.2252, 0.0858, 0.0297, 0.0789, and 0.0856. Therefore, it can be stated that the performance of NN classifier was significantly better than that of other algorithms, apart from LDA.

**Figure 5:**
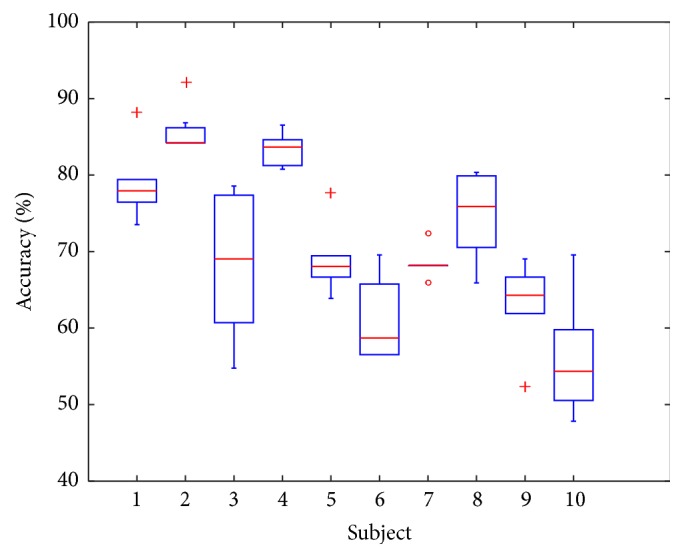
A summary of general accuracies that were obtained for each subject.

In order to evaluate the individual capabilities and suitability of each subject for the use of pilot aiding system based on the principle described in this article, a summary of all accuracies obtained with different classifiers for each subject has been presented in Figure 5. The low variance of results achieved for subjects 1, 2, 4, 5, 7, and 9 suggests that these participants are suitable for work with EEG-based pilot aiding systems. It can be also observed that for subjects 6, 8, and 10 the proper choice of classification algorithm might result in improved performance, while for subject 3 such selection is crucial in order to achieve the best results. It is worth observing that for 10th subject classification accuracies are in general unsatisfactory, which might suggest that either this person is not suitable for work with described systems or the data might have been too noisy due to some unwanted environmental factors.

## 4. Conclusions

In this work a methodology of EEG signals processing and classifier tuning was proposed and evaluated for the purpose of analyzing data containing states of brain activity related to idle but focused anticipation of visual cue and reaction to that cue. Although such methodology has been in use for many classical BCI paradigms, to the best of our knowledge its implementation to the problem posed in this research is a novelty. Classification accuracies obtained during performed tests show the significance of proper selection and fine tuning of classification algorithm. In general case the Neural Network classifier achieved the best mean accuracy, outperforming by almost 5% the LDA and other classifiers by over 8%. However, through the ANOVA tests it was not possible to prove that any differences in means are significant, if all classifiers were considered. This might be attributed to the small number of subjects that participated in the experiment and suggests that for a more reliable and profound analysis, evaluation of proposed methodology and experiment with greater number of participants must be performed.

A very interesting observation was made that, for some subjects, the proposed methodology was not able to find a configuration of parameters that would allow achieving a satisfactory results. This could be attributed to some kind of data corruption; however, a most likely related explanation might be related to the phenomena referred to as* BCI illiteracy* [30]. Accordingly to research and some documented cases, some people are not capable of using BCI (Brain-Computer Interface) systems [30–32]. Such condition must be taken into consideration in the future works and, even more importantly, if such solution as described in this article was to be utilized in real life situations as a part of a pilot aiding system.

Moreover, obtained results proved the possibility of using EEG-based BCI systems in cognitive cockpit solutions. Pilot aiding and reaction enhancing solutions, especially, that are applicable during flight sessions could potentially highly benefit from use of such signals. It must be noted that conducted research was focused mainly on the problem of discrimination between states of brain activity related to idle but focused anticipation of visual cue and reaction to it. Therefore, it should be considered more as an in-depth study of one of the multiple steps of the functional cognitive cockpit system rather than as a description of a complete solution. In order to apply the proposed methods for BCI systems in cognitive cockpit solution it would be necessary to develop automatic methods for the removal of artifacts related to body movements and EMG.

Data recorded for the purposes of this research was acquired using a low-cost and consumer available EEG device with limited number and configuration of electrodes. Despite that, used signals allowed to discriminate between defined classes of brain activity. This validates the potential of utilizing such EEG devices in future work and real life applications. This is a very important conclusion, since professional EEG measurement systems can be very expensive. Most scientifically and clinically used EEG measurement systems provide a great number of electrodes (usually over 60 or even 100). Such approach allows achieving a higher spatial resolution of EEG data. As a result a more accurate and precise conclusions about areas of brain activation can be drawn. However, greater number of measurement electrodes can significantly increase time required for experimental setup and, even more importantly, decrease a comfort of BCI systems and restrict the allowed movement range of subject. Such situation is unacceptable for cognitive cockpit and general pilot monitoring and aiding systems. Therefore, the fact this research proved, that smaller number of electrode channels can be effectively used in such applications, is valuable in terms of practical solutions. Although there are some interesting studies regarding the choice of classification algorithms for the BCI purposes, these are mostly focused on the classical BCI paradigms. To the best of our knowledge a review of classification algorithms in the task of classification of pre- and postevent related activity has not been so far conducted, especially for experiment with low-cost EEG systems. Thanks to the findings of this article a clear information about the choice of the classification method in the proposed methodology of EEG signal analysis was obtained. This will hopefully greatly contribute to the future research on that subject. Achieved results and conclusions drawn from performed experiment will serve as a reference for future works that will be focused not only on digital signal processing and classification of pilot's mental states present during flight session but also on developing of data recording procedures and hardware setup of measurement devices.

## Figures and Tables

**Table 1:** Linear Discriminant Analysis: accuracy of classification achieved for each subject (mean accuracy 73.01%).

Subject	1	2	3	4	5	6	7	8	9	10
Accuracy	79.41%	84.21%	78.57%	82.69%	66.67%	69.57%	68.18%	73.21%	66.67%	60.87%

**Table 2:** *k*-Nearest Neighbors: accuracy of classification achieved for each subject (mean accuracy 69.45%).

Subject	1	2	3	4	5	6	7	8	9	10
Accuracy	79.41%	84.21%	59.52%	80.77%	66.67%	56.52%	68.18%	80.36%	66.67%	52.17%

**Table 3:** Support Vector Machines with linear kernel: accuracy of classification achieved for each subject (mean accuracy 67.29%).

Subject	1	2	3	4	5	6	7	8	9	10
Accuracy	76.47%	84.21%	64.29%	80.77%	63.89%	56.52%	68.18%	69.64%	52.38%	56.52%

**Table 4:** Support Vector Machines with radial basis function kernel: accuracy of classification achieved for each subject (mean accuracy 69.32%).

Subject	1	2	3	4	5	6	7	8	9	10
Accuracy	73.53%	84.21%	73.81%	84.62%	69.44%	56.52%	73.21%	65.91%	61.90%	50.00%

**Table 5:** Random Forest: accuracy of classification achieved for each subject (mean accuracy 68.72%).

Subject	1	2	3	4	5	6	7	8	9	10
Accuracy	76.47%	86.84%	54.76%	84.62%	69.44%	60.87%	65.91%	78.57%	61.90%	47.83%

**Table 6:** Artificial Neural Networks: accuracy of classification achieved for each subject (mean accuracy 77.77%).

Subject	1	2	3	4	5	6	7	8	9	10
Accuracy	88.23%	92.10%	78.57%	86.53%	77.77%	67.39%	68.18%	80.35%	69.04%	69.56%

**Table 7:** Accuracy of classification achieved for each subject.

Classifier	*μ*	*σ*	*Q*_1_	*Q*_3_
LDA	73.01%	7.85%	66.67%	79.41%
kNN	69.45%	11.28%	59.52%	80.36%
SVM_LIN_	67.29%	10.72%	56.52%	76.47%
SVM_RBF_	69.32%	11.12%	61.90%	73.81%
RF	68.72%	12.85%	60.87%	78.57%
NN	77.77%	9.08%	69.04%	86.53%
